# Effects of parent-administered massage-based multimodal interventions on health outcomes in preterm infants: a systematic review and meta-analysis

**DOI:** 10.3389/fpsyg.2026.1922153

**Published:** 2026-10-02

**Authors:** Yan Li, HuanHuan Zhang, Wenyao Ma, Beibei Zou, Wenbo Wu

**Affiliations:** 1 Children’s Health Centre, Ganzhou Nankang Hospital of Traditional Chinese Medicine, Ganzhou, Jiangxi, China; 2 Department of Gynecology, Beijing University of Chinese Medicine, Shenzhen Hospital (Longgang), Shenzhen, Guangdong, China; 3 Orthopedics Rehabilitation Hall, Cangzhou Hospital of Integrated Traditional Chinese and Western Medicine, Cangzhou, Hebei, China; 4 Department of Internal Medicine, Fuzhou Linchuan Hospital of Traditional Chinese Medicine, Fuzhou, Jiangxi, China; 5 Shenzhen Clinical School of Medicine, Guangzhou University of Chinese Medicine, Shenzhen, Guangdong, China

**Keywords:** feeding outcomes, growth, massage, neonatal intensive care, parent-administered intervention, preterm infant

## Abstract

**Background:**

Parent-administered massage-based interventions are increasingly used in family-centered care for preterm infants, but their effects across infant and parental outcomes remain uncertain.

**Objective:**

To systematically evaluate the effects of parent-administered massage-based interventions on growth, feeding, hospitalization, neurodevelopmental, and parental psychological outcomes in preterm infants.

**Methods:**

We conducted a PRISMA 2020-compliant systematic review and meta-analysis of randomized controlled trials. PubMed/MEDLINE, Scopus, and Web of Science were searched from inception to 26 May 2026. Eligible studies compared parent-administered massage alone or combined with tactile, kinesthetic, oral, or other developmental sensory components against routine care or non-massage controls. Random-effects models with restricted maximum-likelihood estimation were used. Parental psychological outcomes were synthesized narratively because of substantial heterogeneity. Risk of bias was assessed with RoB 2, and certainty of evidence with GRADE.

**Results:**

Twenty-four randomized controlled trials were included. Moderate-certainty evidence indicated that massage-based interventions were associated with higher odds of breastfeeding or human-milk feeding at discharge (OR 5.42, 95% CI 2.44–11.94), shorter time to full oral feeding (MD −2.04 days, 95% CI −3.95 to −0.13), and shorter hospital length of stay (MD −4.68 days, 95% CI −7.79 to −1.57). Weight-gain velocity was greater in the primary meta-analysis (MD 5.55 g/day, 95% CI 1.71–9.38), but certainty was low, heterogeneity was very high (I^2^ = 97.87%), significant small-study effects were detected, and the trim-and-fill adjusted estimate was attenuated and no longer statistically significant (MD 3.45 g/day, 95% CI −1.67 to 8.56). Parent-related psychological findings were generally favorable but were supported by low-certainty evidence. Effects on length growth, head-circumference growth, and motor development were not statistically significant; certainty for length and head-circumference growth was very low.

**Conclusion:**

Moderate-certainty evidence suggests that parent-administered massage-based interventions may improve selected feeding outcomes and reduce hospital length of stay in preterm or low-birth-weight infants. The effect on weight-gain velocity remains uncertain because of very high heterogeneity, low certainty of evidence, and possible small-study effects. However, many interventions were multimodal and heterogeneity was substantial; therefore, the findings should be interpreted as effects of parent-delivered developmental-care interventions that include massage rather than massage alone, and the independent effect of massage requires confirmation.

**Systematic review registration:**

https://www.crd.york.ac.uk/PROSPERO/view/CRD420261435482, identifier CRD420261435482.

## Introduction

Preterm birth, defined as birth before 37 completed weeks of gestation, remains a major global child-health challenge. An estimated 13.4 million infants were born preterm in 2020, and complications of prematurity continue to account for a substantial proportion of neonatal and under-five mortality (Liu et al., 2016; Ohuma et al., 2023). Survivors are at increased risk of respiratory, gastrointestinal, neurological, sensory, cognitive, and behavioral complications, as well as longer-term health and developmental sequelae (Mangham et al., 2009; Mwaniki et al., 2012; Saigal and Doyle, 2008). These risks are amplified by prolonged hospitalization, feeding immaturity, and the need for repeated medical procedures, underscoring the importance of safe, scalable, and family-centered interventions that can support early physiological stability and development.

The neonatal intensive care unit (NICU) is essential for survival but differs markedly from the intrauterine sensory environment. Preterm infants may be exposed to bright light, noise, painful procedures, invasive respiratory support, and prolonged parent-infant separation, all of which can affect stress regulation, behavioral organization, feeding readiness, and parent-infant interaction (Als, 1982; Blackburn, 1998; Grunau et al., 2009; Treyvaud, 2014; Xie et al., 2019). After discharge, families may continue to face challenges related to infant care, developmental stimulation, and parental psychological distress. Low-cost interventions that can be taught to parents and continued across hospital and home settings are therefore clinically attractive.

Infant massage is a structured form of tactile and, in some protocols, kinesthetic stimulation. When delivered by parents, massage may provide both direct infant stimulation and an active caregiving role for parents. Proposed mechanisms include increased vagal activity, improved gastrointestinal motility and nutrient utilization, and better sleep–wake and behavioral organization. Massage may also influence stress regulation, although evidence for a consistent reduction in physiological stress markers is limited (Field et al., 2010; Field, 2010; Gunnar and Quevedo, 2008). Parent-administered massage may also enhance caregiver confidence, perceived competence, and emotional bonding, which are relevant to family-centered neonatal care.

Randomized trials have evaluated parent-administered massage-based interventions across outcomes including weight gain, linear growth, head-circumference (HC) growth, feeding progression, hospital length of stay, neurodevelopment, physiological regulation, and parental psychological well-being (Aylward, 2005; Fan et al., 2021; Feijó et al., 2006; Fontana et al., 2018; Fucile et al., 2025; Hwu et al., 2023; Kumar et al., 2013; Lai et al., 2022; Lotfalipour et al., 2019; Mendes and Procianoy, 2008; Procianoy et al., 2010; Zaferani et al., 2021). However, findings remain inconsistent. Study populations, gestational age ranges, intervention timing, session duration, total exposure, use of oil or emollients, provider training, setting, comparator care, and follow-up intervals vary widely. In addition, many protocols combine massage with oral stimulation, kinesthetic stimulation, kangaroo care, visual interaction, or other developmental components, making it difficult to attribute effects to massage alone. These components may act through different pathways. For example, oral stimulation may directly promote sucking and oral-motor readiness, whereas massage may primarily influence autonomic regulation, gastric motility, and behavioral organization.

Previous systematic reviews have suggested potential benefits of infant massage for selected outcomes, particularly weight gain or feeding tolerance, but several were not restricted to parent-administered interventions, did not comprehensively synthesize parental outcomes, or preceded more recent randomized trials (Lu et al., 2020; Seiiedi-Biarag and Mirghafourvand, 2020). Therefore, an updated synthesis focusing on parent-administered massage-based interventions and evaluating both infant- and parent-centered outcomes is warranted.

This systematic review and meta-analysis aimed to systematically evaluate the effects of parent-administered massage-based interventions on growth, feeding, hospitalization, neurodevelopmental, physiological, and parent-related outcomes in preterm or low-birth-weight infants. Accordingly, the review question was: among preterm or low-birth-weight infants, what are the effects of parent-administered massage-based interventions, compared with routine care or other eligible control conditions, on infant and parent outcomes?

## Materials and methods

### Protocol and reporting

This systematic review and meta-analysis was prospectively registered in PROSPERO (CRD420261435482) and was conducted in accordance with the PRISMA 2020 statement. The review question followed the PICOS framework: preterm or low-birth-weight infants as the population; parent-administered massage-based interventions as the intervention; routine care, standard care, minimal handling, or other eligible control conditions as comparators; growth, feeding, hospitalization, neurodevelopmental, physiological, and parent-related outcomes as outcomes; and randomized controlled trials as the eligible study design.

### Literature search and study selection

PubMed/MEDLINE, Scopus, and Web of Science were searched from inception to 26 May 2026. PubMed/MEDLINE was used as the primary database for search-strategy development. Preliminary test searches were conducted in PubMed/MEDLINE to refine the terminology, identify relevant indexing and free-text terms, and confirm retrieval of known eligible studies. The finalized PubMed/MEDLINE strategy was subsequently adapted to the syntax and indexing requirements of Scopus and Web of Science. The final database-specific strategies were checked for syntax and indexing compatibility and tested to confirm retrieval of known eligible studies. The complete database-specific search strategies are provided in Supplementary Table 2. The search strategy combined controlled vocabulary and free-text terms for preterm or low-birth-weight infants, massage or tactile/kinesthetic stimulation, parent-delivered interventions, growth, feeding, neurodevelopmental, physiological, and parental outcomes. The complete keyword blocks and database-specific strategies are provided in Supplementary Tables 1, 2. No publication-year restriction was applied. Reference lists of eligible studies and relevant systematic reviews were manually screened to identify additional records. All retrieved records were imported into EndNote version 25.3.1 for reference management and subsequently exported to Microsoft Excel for screening. Duplicate records were identified using title, author, publication year, DOI, and other available bibliographic fields, followed by manual verification. After duplicate records were removed, two reviewers independently screened titles and abstracts. Full-text reports of potentially eligible studies were then independently assessed against the predefined eligibility criteria. Inter-reviewer agreement for full-text assessment was calculated from the reviewers’ independent pre-consensus decisions using percentage agreement and Cohen’s kappa. Agreement was observed for 430 of 452 full-text reports (95.1%), with a Cohen’s kappa of 0.66. Disagreements were resolved through discussion and, when necessary, consultation with a third reviewer. The selection process is shown in Figure 1. Authors of the included trials were not contacted because the information required for eligibility assessment and quantitative synthesis was available in the published reports or could be derived using established statistical procedures. No otherwise eligible study required exclusion because of unresolved missing information.

**FIGURE 1:**
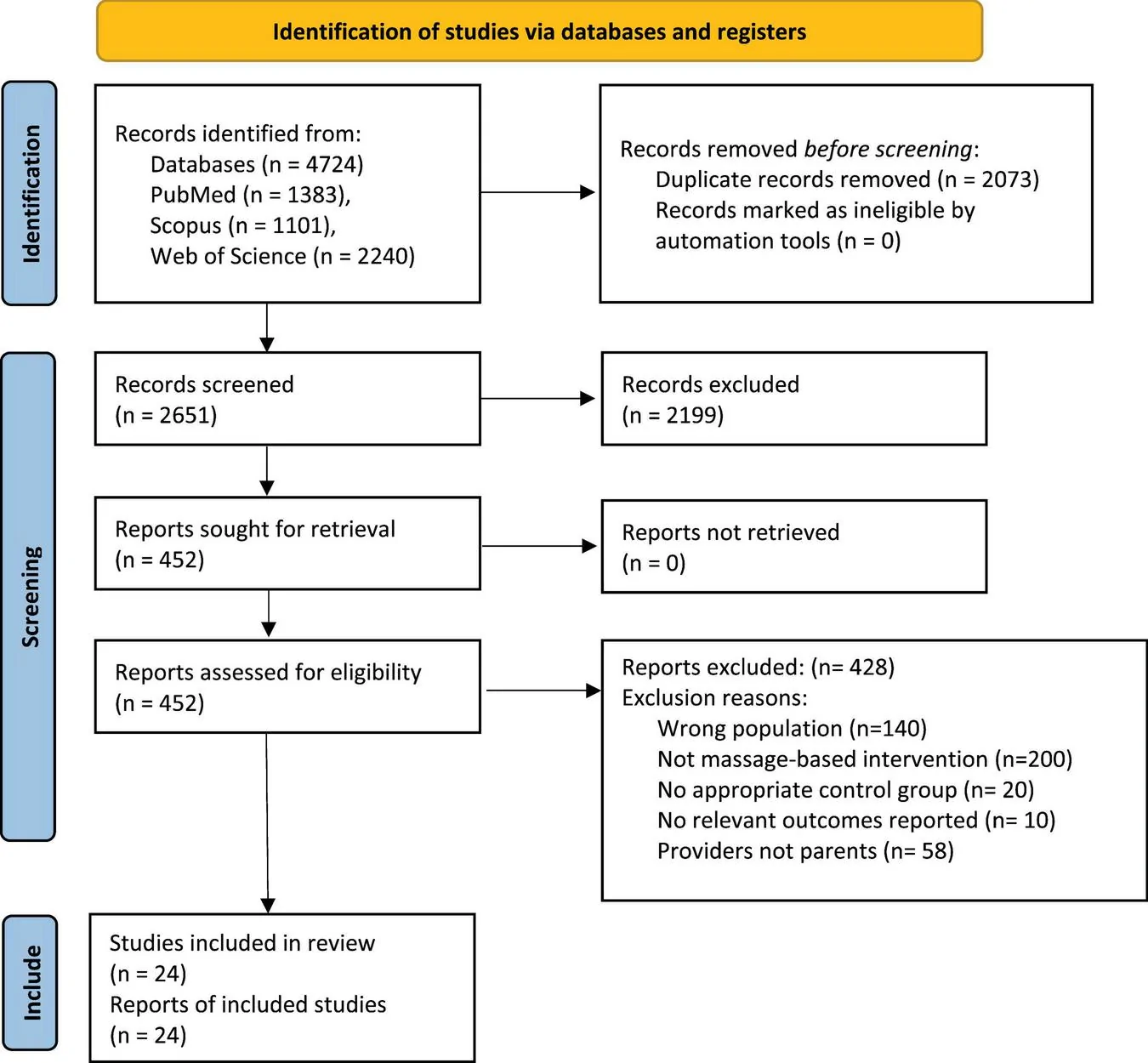
PRISMA 2020 flow diagram of study identification, screening, eligibility assessment, and inclusion.

### Eligibility criteria

Studies were eligible if they: (1) enrolled preterm or low-birth-weight infants; (2) evaluated parent-administered massage-based interventions, including massage alone or massage combined with tactile, kinesthetic, oral, or other developmental sensory components; (3) compared the intervention with routine care, standard care, minimal handling, skin-to-skin care without the additional tactile–kinesthetic component, or another eligible control condition; and (4) reported at least one extractable outcome related to growth, feeding, hospitalization, neurodevelopment, physiological status, or parental outcomes. The main growth outcomes were weight gain or weight-growth velocity, linear growth, and HC growth. Clinical and feeding-related outcomes included breastfeeding status at discharge, time to oral feeding, and length of hospital stay. Developmental outcomes included motor development, whereas parent-related outcomes included stress, anxiety, mood symptoms, attachment, perceived caregiving competence, and self-efficacy. Studies were excluded if they had no eligible comparator, did not include an infant massage-based intervention, reported duplicate or overlapping data without additional relevant outcomes, lacked extractable quantitative data, or were conference abstracts, editorials, narrative reviews, protocols, or non-comparative case reports.

### Data extraction

Two reviewers independently extracted data using a predefined extraction form. Extracted information included first author, publication year, country, study design, sample size, infant characteristics, gestational age, birth weight, sex distribution, postnatal age at intervention, intervention protocol, duration and frequency of massage, total planned sessions, total planned intervention minutes, use of oil, intervention provider, comparator details, follow-up interval, and outcome data. Parent-administered was used as an umbrella term for interventions delivered by a parent or parental caregiver. For study-level characterization, the intervention provider was classified as mother, father, either parent, or parent unspecified, according to the original report. Prescribed intervention duration was considered a protocol characteristic. The prescribed intervention schedule was distinguished from the completed intervention dose whenever adherence information was available. For continuous outcomes, means, standard deviations, and group sample sizes were extracted. When necessary and methodologically appropriate, standard deviations were derived from standard errors, confidence intervals, *P*-values, or other reported dispersion measures. When continuous outcomes were reported as medians with interquartile ranges rather than means and standard deviations, the mean was estimated from the first quartile, median, and third quartile using the method described by Wan et al. (2014), and the standard deviation was approximated from the interquartile range assuming an approximately symmetric distribution. This conversion was required for one included study (González et al., 2024). For outcomes analyzed as change from baseline, the change mean was calculated as the estimated post-intervention mean minus the estimated baseline mean. When the standard deviation of the change was not reported, it was derived from the estimated baseline and post-intervention standard deviations using the standard change-score formula with an assumed baseline–follow-up correlation coefficient of 0.50. These conversions were applied only when the available quartile data were considered sufficiently compatible with an approximately symmetric distribution.

For dichotomous outcomes, the number of events and non-events in each group was extracted. Any discrepancies between reviewers were resolved through discussion and, when necessary, by consultation with a third reviewer.

The direction of effects was harmonized before analysis. For growth outcomes and breastfeeding or human-milk feeding at discharge, positive effect estimates favored massage-based interventions. For time-dependent or higher-burden outcomes, including time to full oral feeding, hospital length of stay, and psychological distress, negative effect estimates favored massage-based interventions.

Inter-reviewer agreement for data extraction was calculated from the reviewers’ independent pre-consensus extraction forms. The reviewers agreed on 97.3% of extracted data items. Discrepancies were resolved through discussion and, when necessary, consultation with a third reviewer.

### Outcome classification

Outcome domains were prespecified and grouped into clinically coherent categories before synthesis to ensure consistency across studies and facilitate interpretation of the pooled estimates. The growth domain included weight gain or weight-growth velocity, linear growth, and HC growth. Because weight was the most consistently reported growth endpoint, it was selected as the primary outcome for the main quantitative synthesis, subgroup analyses, meta-regression, sensitivity analysis, and publication-bias assessment. All weight analyses were conducted using mean difference. The feeding and hospital-course domain included breastfeeding or human-milk feeding at discharge, time to oral feeding, and hospital length of stay. Breastfeeding or human-milk feeding at discharge was analyzed as a dichotomous outcome, whereas time to oral feeding and hospital stay were analyzed as continuous outcomes in days. Breastfeeding or human-milk feeding at discharge was classified as exclusive or non-exclusive when this distinction was reported by the original study. The developmental domain included motor-development outcomes. Motor development was analyzed using standardized mean differences when different developmental scales were used across studies. Parent-related outcomes were classified according to the construct assessed, including stress, anxiety, mood symptoms, attachment, perceived competence, and caregiving self-efficacy. Because these constructs, measurement instruments, scoring directions, and assessment time points differed substantially, parent-related outcomes were synthesized narratively rather than combined into a single pooled estimate.

### Risk-of-bias assessment and certainty of the evidence

Two reviewers independently assessed risk of bias using the revised Cochrane Risk of Bias 2 (RoB 2) tool for randomized controlled trials. The five domains were bias arising from the randomization process, deviations from intended interventions, missing outcome data, measurement of the outcome, and selection of the reported result. Each domain and the overall judgment were rated as low risk, some concerns, or high risk according to RoB 2 guidance. Inter-reviewer agreement was calculated from the reviewers’ independent pre-consensus judgments and was 83.3%. Disagreements were resolved through discussion or consultation with a third reviewer.

The certainty of evidence for each major outcome was assessed using the GRADE framework. Randomized trial evidence was initially rated as high certainty and was downgraded, when appropriate, for risk of bias, inconsistency, indirectness, imprecision, and publication bias. Certainty was categorized as high, moderate, low, or very low (Prasad, 2024).

### Statistical analysis

Meta-analyses were conducted when at least two studies reported sufficiently comparable outcome data. Random-effects models were used throughout because clinical and methodological heterogeneity was expected across studies. Between-study variance was estimated using restricted maximum likelihood, and pooled estimates were reported with 95% confidence intervals.

Continuous outcomes measured on the same scale were summarized using mean differences (MDs). Weight-gain velocity, length-growth velocity, HC growth velocity, time to full oral feeding, and hospital length of stay were analyzed as MDs when comparable metrics were available. Outcome-assessment intervals were examined before pooling. For each study contributing to the weight-gain, length-growth, or head-circumference-growth velocity meta-analysis, we extracted the interval over which growth was assessed, including the first and last measurement time points where reported. When growth velocity was not directly reported but sufficient data were available, it was calculated as the difference between the first and last measured values divided by the duration of the corresponding assessment interval and expressed as g/day for weight and cm/day for length and head circumference. Thus, growth outcomes were normalized to a daily rate according to the study-specific assessment interval. Variation in assessment-interval duration was considered when interpreting between-study heterogeneity. Continuous outcomes measured using different scales were summarized using standardized mean differences (SMDs). Dichotomous outcomes were synthesized as log odds ratios and back-transformed to odds ratios (ORs) for interpretation.

Heterogeneity was assessed using Cochran’s Q test, I^2^, H^2^, and tau^2^. For the primary weight outcome, prespecified subgroup analyses were performed according to oil or emollient use and intervention provider. Studies were grouped according to whether the intervention was delivered specifically by mothers only or could be delivered by either/both parents. A categorical subgroup analysis using a <32 versus ≥32-week gestational-age threshold was intended. However, several included trials enrolled infants whose gestational-age ranges crossed this threshold and did not report intervention effects separately for these gestational-age categories. Assigning such studies to a subgroup according to the study-level mean gestational age would therefore risk ecological misclassification. Gestational age was instead examined as a continuous study-level moderator in random-effects meta-regression. Additional exploratory univariable random-effects meta-regression examined selected study-level covariates, including sex distribution, postnatal age at intervention, minutes per session, planned number of sessions, and total planned intervention minutes. These analyses were interpreted as hypothesis-generating because they were based on aggregate study-level data and a limited number of studies.

A cumulative meta-analysis ordered studies by intervention or outcome-assessment interval was used to evaluate how the weight-gain effect evolved across follow-up duration. To explore whether the accumulated evidence changed over calendar time, an exploratory cumulative meta-analysis was additionally performed for the primary weight-gain-velocity outcome, with studies entered chronologically according to publication year. Publication year was considered an indicator of the chronological evolution of the evidence and was not interpreted as a proxy for implementation of Family Integrated Care (FICare).

Leave-one-out sensitivity analysis assessed whether the primary result was driven by any single study. Small-study effects were evaluated for the primary weight outcome by visual funnel-plot inspection, Egger’s regression test, Begg’s rank-correlation test, and trim-and-fill analysis. The trim-and-fill analysis was interpreted as a sensitivity analysis and not as a definitive correction for publication bias. When an adequate number of studies was available. A two-sided *P* < 0.05 was considered statistically significant.

## Results

### Study selection

The study-selection process is presented in Figure 1. Inter-reviewer agreement for full-text assessment was 95.1% (430/452 reports), with a Cohen’s kappa of 0.66, based on the reviewers’ independent pre-consensus decisions. Disagreements were resolved through discussion, and no study was excluded because of author non-response.

### Characteristics of included studies

The characteristics of the included studies are summarized in Table 1. Twenty-four randomized controlled trials involving preterm or low-birth-weight infants met the eligibility criteria. Studies were conducted across 12 countries predominantly enrolled infants born between approximately 25 weeks and less than 37 completed weeks of gestation. Interventions consisted of parent-administered massage-based protocols delivered with or without oil/emollient in NICU, hospital, post-discharge, or home settings. Several interventions combined massage with tactile, kinesthetic, oral/sensorimotor, visual, kangaroo-care, or other developmental components Comparator groups received routine or standard care, minimal handling, attention control, skin-to-skin care without the additional tactile–kinesthetic component, or another eligible comparison condition. Most interventions were delivered by mothers; however, some studies permitted either parent to provide the intervention or did not clearly specify the parent involved. The prescribed intervention schedule, completed dose, and source of adherence information are reported separately in Table 1 when available. Outcomes were grouped into growth, feeding and hospitalization, neurodevelopmental, physiological, and parental psychological domains.

**TABLE 1:** Characteristics of included randomized controlled trials evaluating parent-administered massage-based interventions in preterm or low-birth-weight infants.

Study (first author, year, country)	Study design	Participants	GA intervention (weeks)	GA control (weeks)	Oil used	Intervention provider	Setting	Intervention details	Intervention duration	Outcomes
Fan et al., 2021, China	RCT	Early preterm infants (28+0 to 31+6 wks) (73 intervention, 33 control)	30.13 ± 1.56	30.35 ± 1.92	Not mentioned	Parents (trained by researchers)	Home-based after NICU discharge	Hearing/vision training + whole-body massage + kangaroo care + integrated hearing-vision training	Daily × 30 days	TIMP motor score, DQ, weight, length, head circumference
Fucile et al., 2025, Canada	RCT	Preterm infants in NICU, not on full oral feeds (GA ∼31–32 wks)	31 ± 2.6	32 ± 1.8	No	Parents (mothers 91%)	NICU bedside	PASI: oral stroking + non-nutritive sucking + trunk/limb stroking	15 min once daily × 10 days (within 14 days)	Days to full oral feeds, direct breastfeeding or human-milk feeding at discharge, TIMP score, parent satisfaction
Kumar et al., 2013, India	RCT	Preterm neonates <1800 g (GA ∼32.6–32.9 wks)	32.9 ± 1.4	32.6 ± 1.4	Yes – sunflower oil (2.5 mL/kg)	Mothers (trained; initial sessions by staff)	NICU + home after discharge	Sunflower oil massage, moderate pressure, 20 strokes/area, standardized technique	10 min × 4 times/day (40 min/day) × 28 days	Weight gain, length, head circumference, triglycerides
Mokaberian and Faez, 2020, Iran	RCT	40 preterm infants + mothers in NICU (20 intervention, 20 control; GA 32–<37 wks)	32–37	32–37	Yes – almond oil	Mothers (trained by researcher)	NICU bedside (mother-performed)	Tactile-kinesthetic stimulation (3 phases): stroking (side-lying) + passive limb movements (supine) + stroking; almond oil	20 min × 3/day × 10 consecutive days	Maternal-infant attachment (MPAS)
Keshavarz et al., 2012, Iran	RCT	60 preterm infants post-NICU discharge (30 per group; GA 32– < 37 wks)	32–37	32–37	Yes – almond oil	Mothers (trained)	Home after NICU discharge	Touch therapy: 2 tactile phases (8 min each) + 1 kinesthetic phase (4 min); almond oil; continued to 40 wks corrected age	20 min × 3/day until 40 wks corrected age	Daily weight gain
Fontana et al., 2018, Italy	RCT	70 very preterm infants (25+0 to 29+6 wks); 57 analyzed at discharge	25+0 to 29+6	25+0 to 29+6	Not reported (moderate-pressure stroking)	Parents (mainly mothers) + researcher (PremieStart)	NICU (+ 1 post-discharge session)	PremieStart parental training + parent massage (back, slow tactile) + visual interaction (black-white toy/face)	Massage 2 × /day; visual 1 × /day until discharge; PremieStart 8 sessions + 1 post-discharge	Human milk feeding at discharge
Fontana et al., 2020, Italy	RCT	70 very preterm (25+0 to 29+6 wks); 57 assessed at term equivalent age	25+0 to 29+6	25+0 to 29+6	Not reported	Parents (mainly mothers)	NICU until term equivalent age	PremieStart + parent massage (back, 10 min tactile 2 × /day from ∼32 wks) + visual interaction (1 × /day from 34 wks)	Massage 2 × /day + visual 1 × /day from 32 to 34 wks PMA until TEA	Better visual function at TEA; ocular motility, tracking, attention
Hwu et al., 2023, Taiwan	RCT	61 preterm infants (GA 35.1 ± 1.5 wks, BW 1500–2500 g) + parents; home after discharge	35.1 ± 1.5	35.1 ± 1.5	Yes – plant-based (sunflower + apricot + sweet almond)	Both parents (mother + father, trained)	Home after NICU discharge	Modified Field protocol: tactile (prone, 5 body parts) + kinesthetic (supine limb flexion/extension) + repeat tactile; oil used	15 min × 2/day (once each parent) × 7 days/wk × 12 weeks	Weight gain at 4, 8, 12 weeks; parental stress (PSI-SF) at 8 and 12 weeks parental attachment
Jamshaid et al., 2021, Pakistan	RCT	Preterm and LBW neonates (GA 28–37 wks, BW 1.5–2.5 kg)	28–37	28–37	Yes – sunflower oil (10 mL/kg/day)	Mothers (at home)	Home / community after enrollment	Sunflower oil massage, twice daily	2 applications/day × 2 months	Weight gain, length gain
Pramitha et al., 2026, Indonesia	RCT	56 mothers of preterm infants in NICU (28 per group)	<37	<37	Not mentioned	Mothers (trained by certified instructor)	NICU during hospitalization	Infant massage per 2022 Indonesian Pediatric Society module (5–10 min/session)	5–10 min × 2/day (morning + evening) × 7 days	Maternal self-confidence (PMP S-E):
Zaferani et al., 2021, Iran	RCT	80 mothers of preterm infants in NICU (40 per group)	Preterm (NICU required)	Preterm (NICU required)	Not mentioned	Mothers (trained with doll + brochure)	NICU from admission to discharge	Positive Touch: preparation; initiating touch; containment; pacing; kangaroo care; letting go	Variable (mean ∼24–27 days hospital stay); performed during visits	Maternal total mood disturbance; POMS subscales
Lai et al., 2022, Australia	RCT	60 very preterm (28–32+6 wks), low-risk brain injury; 45 analyzed for EEG	28 to 32+6 (start at 34 wks PCA)	28 to 32+6 (standard care from 34 wks PCA)	Yes – cold-pressed fruit/vegetable oil	Mothers (trained)	NICU / hospital (34 wks PCA to 40 wks CA)	Modified field: tactile (prone, moderate pressure stroking 6 areas) + kinesthetic (supine limb flexion/extension); oil used	15 min × 2/day from 34 wks PCA until 40 wks corrected age	Primary beta GRP; central alpha GRP, EEG
Zhang and Wang, 2019, China	RCT	112 stable preterm (GA 32–34 wks); 58 massage, 54 control	32–34 (start when stable)	32–34	Not explicitly mentioned	Mothers (trained by specialist)	NICU bedside/incubator	Modified field: tactile (prone, 5 steps moderate pressure) + kinesthetic (supine limb movements); 1 h before feeding	15 min × 2/day × 2 consecutive weeks (28 sessions)	Weight, height, head circumference
Procianoy et al., 2010, Brazil	RCT	73 VLBW infants (≤32 wks, BW 750–1500 g); followed to 2 yrs corrected age	30.0 ± 1.55	29.7 ± 1.62	No (direct skin contact)	Mothers (trained)	NICU bedside (until discharge)	Tactile-kinesthetic stimulation (face + limbs stroking + passive joint movements); all also received skin-to-skin care	15 min × 4/day (every 6 h) from 48 h of life until discharge	Mental development index at 2 yrs Psychomotor Index
Mendes and Procianoy, 2008, Brazil	RCT	104 VLBW infants (≤32 wks, BW 750–1500 g); 93 completed to discharge	29.7 ± 1.6	29.4 ± 1.6	No (direct skin tactile-kinesthetic)	Mothers (trained)	NICU bedside/incubator	Tactile-kinesthetic (face/limbs stroking + passive joint flexion/extension); all received skin-to-skin care	15 min × 4/day (every 6 h) from 48 h until discharge	Hospital stay; late-onset sepsis
Holditch-Davis et al., 2014, USA	RCT (3-group longitudinal)	240 preterm infants (<1750 g) + mothers from 4 hospitals; followed to 12 mo corrected age	∼27.0–27.4 (overall)	∼27.4	Not mentioned (moderate stroking for ATVV)	Mothers (trained + weekly supervision)	NICU + home until 2 mo corrected age	ATVV (auditory-tactile-visual-vestibular) or Kangaroo Care vs. attention control	≥15 min/session, ≥3 × /wk (preferably daily) during NICU + home to 2 mo CA	Maternal distress; worry, infant social/development; HOME scores
White-Traut and Nelson, 1988, USA	RCT (3-group)	33 mother-infant pairs (preterm 28–35 wks); feeding interaction assessed pre-discharge	RISS: 33.91; Talking: 34.18; Control: 33.18	33.18	Not mentioned	Mothers (taught by researcher)	Special care nursery bedside (pre-discharge)	RISS: cephalocaudal massage (10 min) + rocking (5 min) + talking + eye contact vs. talking only vs. routine care	15 min sessions at 24–36 h, 37–48 h, 49–60 h, 61–72 h post-birth (some only 3 sessions)	Sensitivity to cues; cognitive growth; total behavior scores
Matricardi et al., 2013, Italy	RCT	42 parents (21 mother-father couples) of very preterm (≤32 wks) in NICU	29.35 ± 1.99	29.10 ± 1.87	Yes (moderate pressure stroking + kinesthetic)	Both parents (trained and supervised by PT)	NICU bedside (under PT supervision)	Standard support + 8 sessions: joint observation (NNNS-based) + parent-delivered infant massage with oil	8 × 1-h sessions (31–36 wks PMA); massage in sessions 4–6	Parental stress at discharge on IBA and PRA scales
Feijó et al., 2006, USA	RCT	40 mothers + preterm infants about to be discharged from NICU	Performing: ∼28; Observing: ∼30 (at birth)	∼29 overall	No	Mothers themselves (Performing group)	NICU bedside (single session pre-discharge)	Mothers performed 4-min moderate-pressure stroking massage on own infant vs. only observed researcher doing it	Single 8-min session (two 4-min segments) just before discharge	Depressed mood; anxiety reduction
González et al., 2024, Mexico	RCT	70 premature neonates (28–36 wks) in nursery unit; hemodynamically stable	Median 33 (IQR 31–34)	Median 33 (IQR 29–34)	No (explicitly without oil/lubricant)	Parents (usually mother) under nurse supervision	Nursery unit / incubator bedside	Vimala massage (Hindu/Swedish/reflexology/ yoga mix): face; arms; chest; abdomen; legs; back; moderate pressure, 5 repeats each area	15–20 min × 2/day × 5 consecutive days	Weight gain; Heart rate; Respiratory rate; salivary cortisol
Fallah et al., 2013, Iran	RCT	Medically stable preterm LBW neonates (GA ∼34.5–34.6 wks)	34.6 ± 1.96	34.5 ± 1.26	Yes – sunflower oil (10 mL/kg/day)	Mothers (trained by researcher)	NICU + home after discharge	Moderate-pressure tactile + kinesthetic massage with sunflower oil	10 min × 3/day × 14 consecutive days	Weight, height, head circumference, adverse events
Whipple, 2000, USA	RCT	20 preterm LBW infants (<37 wks, BW ≤ 2500 g) + parents in NICU	∼32–33 corrected (range 25–36)	∼32–33 corrected	No	Parents (mothers ± fathers; trained by music therapist)	NICU (visits) + home follow-up	Parent training in music + multimodal stimulation (stroking cephalocaudal + rocking + eye contact) + overstimulation recognition	∼1 h total training; apply during daily care and stress situations (max 60 min/day stimulation)	Infant stress behaviors; parent responses; Hospital stay; weight gain
Shoghi et al., 2018, Iran	RCT	Primiparous mothers + late preterm newborns (GA 34– < 37 wks) in NICU	34–37	34–37	No	Mothers (primiparous)	NICU at referral hospital (bedside)	Head-to-toe massage by mothers using standardized booklet guidelines (naked or diapered infant)	15 min × 3/day × 5 days (started day 5–7 of hospitalization)	Mother-infant attachment behaviors (Avant scale)
Gonzalez et al., 2009, Mexico	RCT	Stable preterm infants in neonatal unit (GA ∼31.4–31.7 wks at birth)	33.5 ± 1.3 at entry	33.1 ± 1.7 at entry	No	Parents (instructed in Vimala massage)	Neonatal unit / hospital	Standardized Vimala massage (face, chest, abdomen, limbs, back) with moderate pressure strokes	15 min × 3/day × 10 consecutive days	Weight gain, head circumference, caloric intake, length of hospitalization

Values are presented as mean ± SD unless otherwise specified. Gestational age refers to age at birth unless explicitly described as postmenstrual, postconceptional, corrected, or term-equivalent age. Several interventions combined massage with other sensory, kinesthetic, oral, visual, kangaroo-care, music, or developmental components; therefore, intervention effects should be interpreted as effects of parent-administered massage-based interventions rather than massage alone; ATVV, auditory, tactile, visual, and vestibular stimulation; BW, birth weight; CA, corrected age; DQ, developmental quotient; EEG, electroencephalography; GA, gestational age; GRP, global relative power; IBA, infant behavior assessment; IQR, interquartile range; LBW, low birth weight; MPAS, Maternal Postnatal Attachment Scale; NICU, neonatal intensive care unit; NNNS, Neonatal Intensive Care Unit Network Neurobehavioral Scale; PASI, parent-administered sensorimotor intervention; PCA, postconceptional age; PMA, postmenstrual age; PMP S-E, perceived maternal parenting self-efficacy; POMS, Profile of Mood States; PRA, parent-reported anxiety; PSI-SF, Parenting Stress Index-Short Form; PT, physiotherapist; RCT, randomized controlled trial; RISS, rocking and infant stimulation/stimulation sequence; TEA, term-equivalent age; TIMP, Test of Infant Motor Performance; VLBW, very low birth weight; wks, weeks.

### Risk of bias assessment

The risk-of-bias assessment is summarized in Supplementary Table 3. Overall risk-of-bias judgments were either low risk or some concerns. Most studies were judged to have a low risk of bias for the randomization process, missing outcome data, and selection of the reported results. The most frequent concerns related to deviations from intended interventions and measurement of outcomes, primarily because participant and caregiver blinding was not feasible in parent-administered massage interventions. No study was rated as having a high overall risk of bias. Overall, eight studies were rated as having a low overall risk of bias, whereas the remaining studies were judged to have some concerns.

### Certainty of the evidence

The certainty of evidence for each outcome was evaluated using the GRADE approach and is summarized in Supplementary Table 4. Evidence was rated as moderate for breastfeeding or human-milk feeding at discharge, time to full oral feeding, and hospital length of stay. The certainty of evidence for weight-gain velocity was rated as low because of very serious inconsistency and suspected publication bias. The certainty of evidence for parent-related psychological outcomes was also rated as low because of risk-of-bias concerns, substantial clinical and measurement heterogeneity, and imprecision. Evidence for length growth velocity and head-circumference growth was rated as very low because of serious inconsistency and imprecision.

### Growth outcomes

Massage-based interventions were associated with greater weight-gain velocity than comparator conditions (10 studies; MD 5.55 g/day, 95% CI 1.71–9.38; *P* = 0.005), although between-study heterogeneity was very high (I^2^ = 97.87%). In contrast, the pooled effects on length growth velocity (5 studies; MD 0.01 cm/day, 95% CI −0.01 to 0.03; *P* = 0.46; I^2^ = 86.74%) and head-circumference growth velocity (7 studies; MD 0.00 cm/day, 95% CI −0.01 to 0.01; *P* = 0.71; I^2^ = 51.70%) were not statistically significant (Figures 2A–C). The assessment interval for growth velocity varied across studies. However, weight, length, and head-circumference changes were expressed as daily velocities based on the corresponding study-specific assessment interval, allowing outcomes measured over different durations to be analyzed on a common time scale. Nevertheless, differences in assessment-interval duration may have contributed to the observed between-study heterogeneity.

**FIGURE 2:**
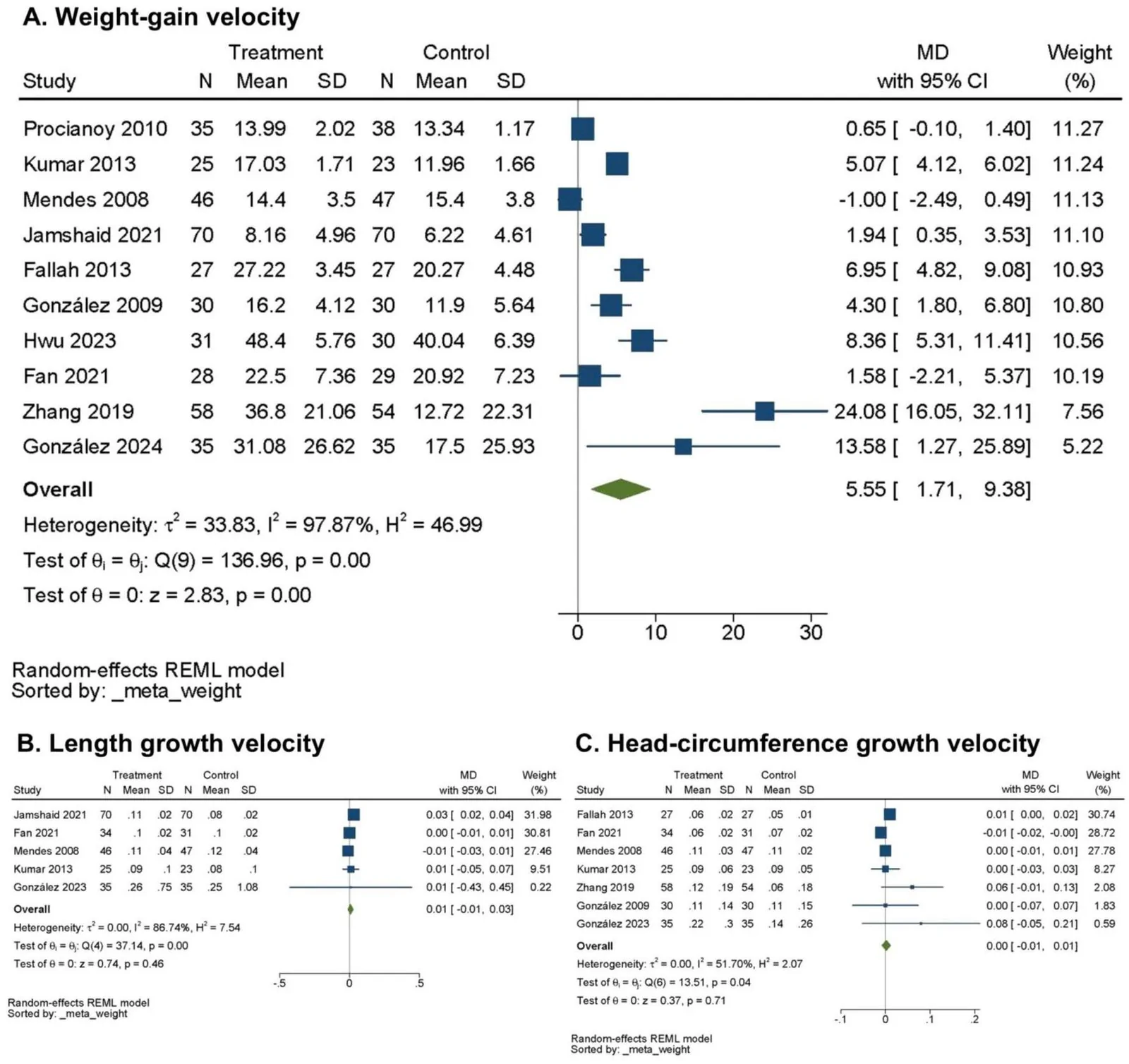
Random-effects meta-analyses of infant growth outcomes. **(A)** Weight-gain velocity, pooled as a mean difference in g/day. **(B)** Length growth velocity, pooled as a mean difference in cm/day. **(C)** Head-circumference growth velocity, pooled as a mean difference in cm/day. Squares represent study-specific estimates, horizontal lines represent 95% confidence intervals, and diamonds represent pooled estimates.

### Feeding and hospital outcomes

Massage-based interventions were associated with higher odds of breastfeeding or human-milk feeding at discharge (2 studies; log OR 1.69, 95% CI 0.89–2.48; *P* < 0.001; I^2^ = 0.00%), corresponding to an OR of 5.42 (95% CI 2.44–11.94). Massage-based interventions also shortened time to full oral feeding (3 studies; MD −2.04 days, 95% CI −3.95 to −0.13; *P* = 0.04; I^2^ = 46.78%) and shorter hospital length of stay (3 studies; MD −4.68 days, 95% CI −7.79 to −1.57; *P* < 0.01; I^2^ = 0.00%) compared with control conditions (Figures 3A–C). The original studies differed in their feeding definitions, including direct breastfeeding and human-milk feeding, and did not consistently specify whether feeding was exclusive or non-exclusive. Accordingly, the pooled estimate represents the study-defined presence of breastfeeding or human-milk feeding at discharge.

**FIGURE 3:**
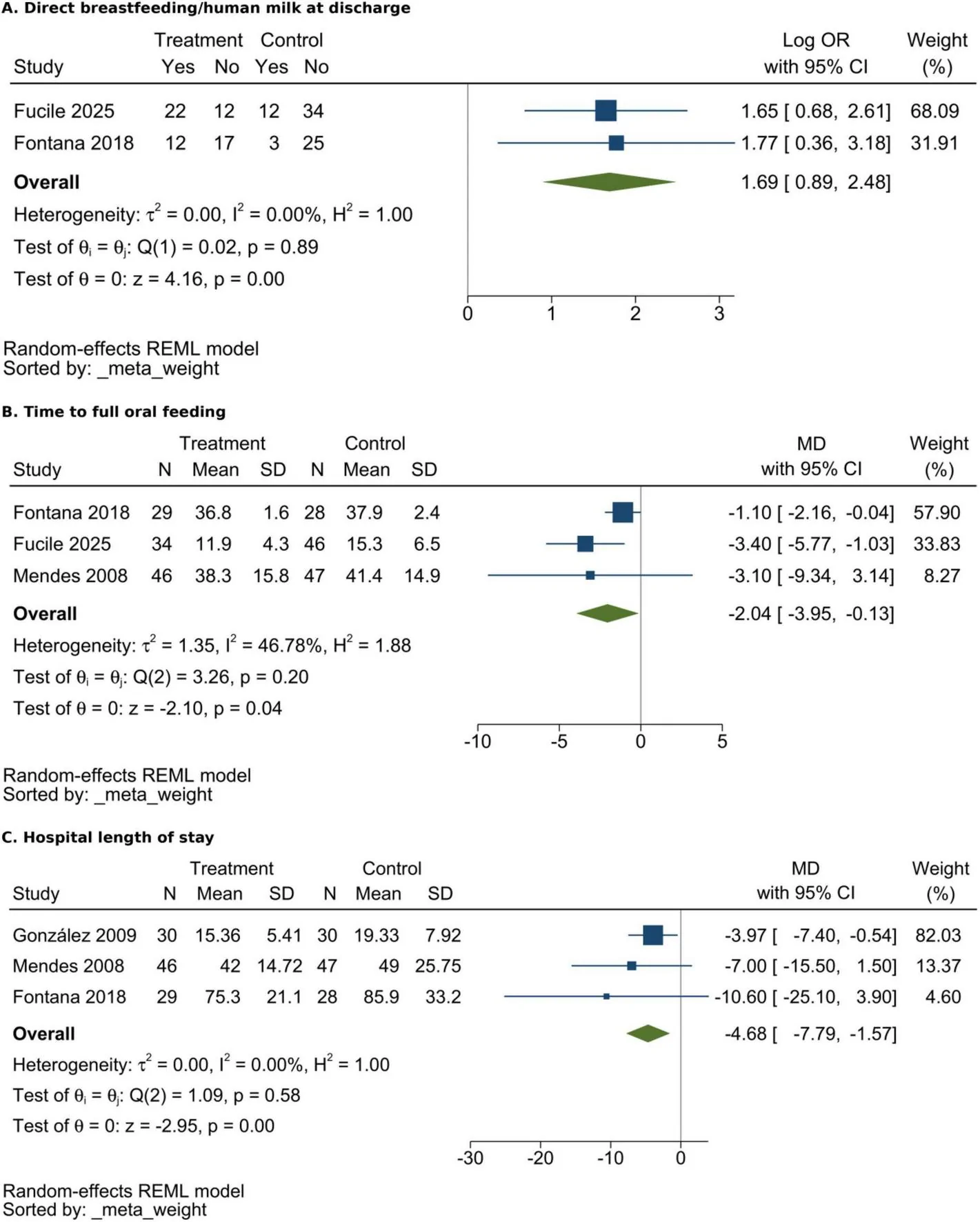
Random-effects meta-analyses of feeding and hospital-course outcomes. **(A)** Direct breastfeeding or human-milk feeding at discharge, pooled as log odds ratios and interpreted as odds ratios in the text. **(B)** Time to full oral feeding in days. **(C)** Hospital length of stay in days. For panels **(B,C)**, negative values favor massage-based interventions.

### Developmental and parent-related outcomes

Parent-related psychological outcomes were synthesized narratively because the included studies assessed different constructs, including parental stress, anxiety, mood disturbance, attachment, and caregiving self-efficacy, using different instruments and assessment time points. Outcomes included parental stress, anxiety and mood symptoms, parent–infant attachment, perceived caregiving competence, and self-efficacy. Assessments were conducted at different stages, including during hospitalization, around discharge, and during post-discharge follow-up. Instruments also varied across studies, including the Profile of Mood States (POMS), Parenting Stress Index–Short Form (PSI-SF), Maternal Postnatal Attachment Scale (MPAS), and measures of perceived maternal parenting self-efficacy. Findings generally favored the intervention in some domains; however, the direction and magnitude of the results varied across constructs, scales, and assessment periods. Accordingly, no common summary effect was considered clinically meaningful.

### Subgroup analyses for weight gain

Because weight gain was the most frequently reported and statistically significant growth outcome, it was selected as the primary outcome for additional subgroup analyses. Studies using oil or emollient during massage showed a larger pooled effect (MD 1.57, 95% CI 0.54–2.59; *P* < 0.001; I^2^ = 92.94%) than studies without reported oil use (MD 0.47, 95% CI 0.06–0.87; *P* = 0.02; I^2^ = 78.51%). The test for subgroup differences was borderline statistically significant (Qb = 3.83; *P* = 0.05), suggesting that oil use may partly modify the weight-gain response (Figure 4A). When studies were stratified by intervention provider, the pooled effect was significant both in studies in which the intervention was delivered specifically by mothers only (MD 1.01, 95% CI 0.12–1.91; *P* = 0.03; I^2^ = 95.61%) and in studies permitting either parent or both parents to deliver the intervention (MD 0.73, 95% CI 0.25–1.20; *p* < 0.01; I^2^ = 70.50%). There was no evidence of a significant difference between provider subgroups (Qb = 0.31; *P* = 0.58); however, the small number of studies within each subgroup limits interpretation (Figure 4B).

**FIGURE 4:**
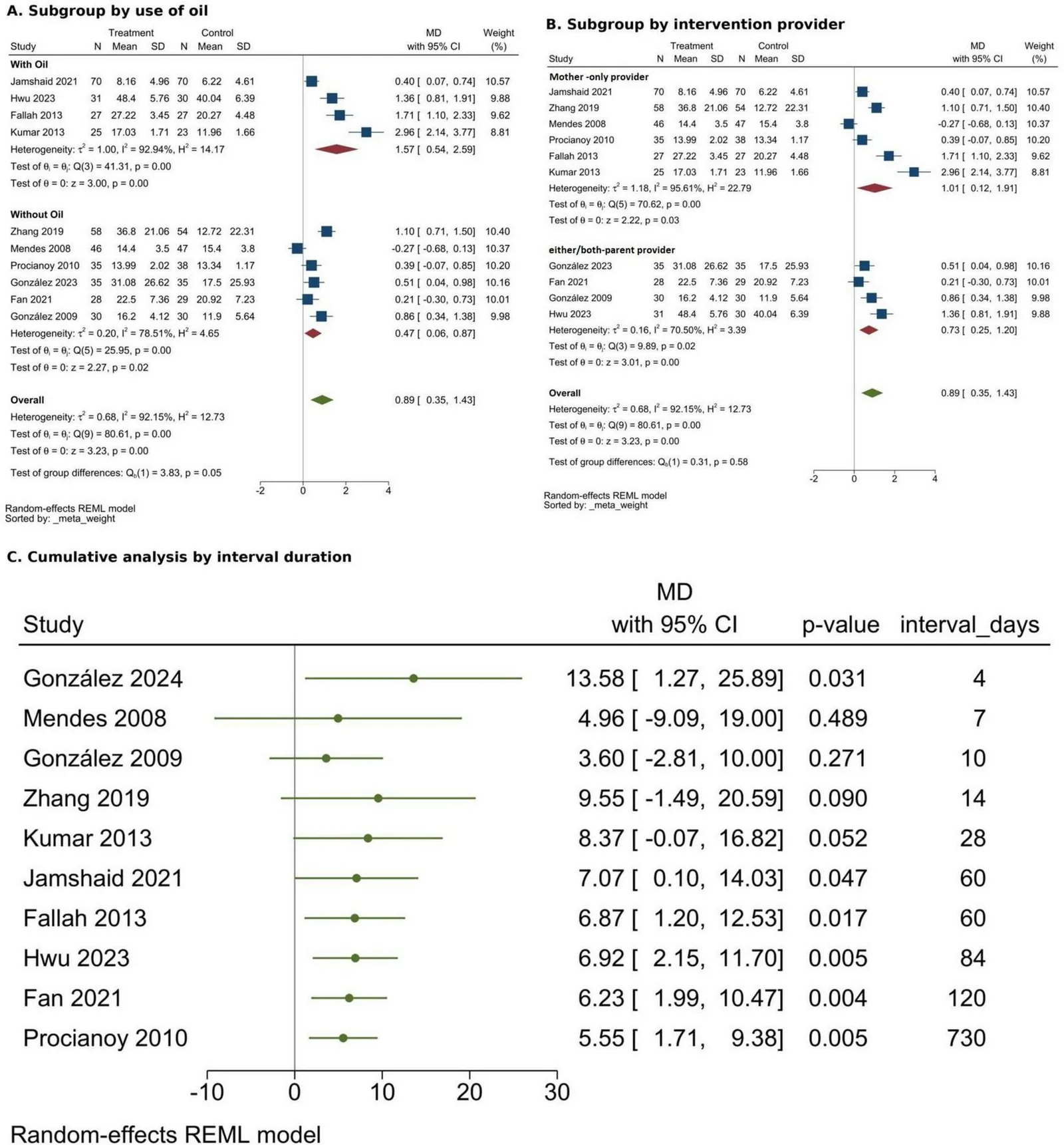
Subgroup and interval-based analyses for the primary weight-gain outcome. **(A)** Standardized subgroup analysis according to oil/emollient use. **(B)** Standardized subgroup analysis according to intervention provider. **(C)** Cumulative meta-analysis ordered by intervention or outcome-assessment interval, reported as mean differences for weight-gain velocity.

### Interval-based cumulative analysis

An exploratory cumulative meta-analysis ordered by the interval between intervention initiation and outcome assessment examined how the Estimates based on studies with shorter assessment intervals were unstable. After studies with assessment intervals of 60 days or longer were added, the cumulative estimate remained statistically significant. The final cumulative estimate remained significant after inclusion of all available interval strata (MD 5.55 g/day, 95% CI 1.71–9.38; *P* = 0.005) (Figure 4C). This pattern should not be interpreted as evidence that longer intervention or follow-up duration causes a larger or more stable effect, because the analysis compared different studies rather than within-study time points.

### Temporal cumulative analysis

An exploratory cumulative meta-analysis ordered by publication year showed that the estimated effect on weight-gain velocity evolved as evidence accumulated over time. The earliest cumulative estimates were imprecise and not statistically significant. The cumulative estimate became statistically significant after inclusion of the 2013 evidence (MD 3.12 g/day, 95% CI 0.24–6.00; *P* = 0.033), although uncertainty remained and the estimate was again non-significant after addition of the 2019 study (MD 6.02 g/day, 95% CI −0.25 to 12.30; *P* = 0.060). With the subsequent 2021–2023 studies, the cumulative estimate remained positive and ultimately reached MD 5.55 g/day (95% CI 1.71–9.38; *P* = 0.005) (Supplementary Figure 2). These findings indicate evolution in the accumulated evidence over time but do not establish a specific temporal change attributable to changes in neonatal care.

### Meta-regression

Random-effects meta-regression for the standardized weight-gain effect showed no significant association with female-to-male ratio (coefficient −0.421, 95% CI −1.860 to 1.018; *P* = 0.566; R^2^ = 0.00%), total planned massage minutes (coefficient −0.00027, 95% CI −0.00092 to 0.00038; *P* = 0.419; R^2^ = 0.00%), or total planned sessions (coefficient −0.0012, 95% CI −0.0101 to 0.0077; *P* = 0.789; R^2^ = 0.00%). Higher mean gestational age was associated with a larger standardized weight-gain effect (coefficient 0.287 per additional gestational week, 95% CI 0.063–0.510; *P* = 0.012; R^2^ = 47.43%), whereas longer minutes per session was inversely associated with the effect size (coefficient −0.236 per minute, 95% CI −0.424 to −0.048; *P* = 0.014; R^2^ = 45.27%). Mean postnatal age was inversely associated with the effect estimate (coefficient −0.155 per day, 95% CI −0.208 to −0.101; *P* < 0.001; R^2^ = 99.87%); however, this model included only three studies and is too unstable to support clinical or causal interpretation. Residual heterogeneity remained significant in most models, supporting cautious interpretation of these moderator findings. Overall, the meta-regression findings should be regarded solely as hypothesis-generating and should not be used to define the optimal timing, session duration, or target gestational age for clinical practice.

### Sensitivity analyses and small-study effects

The leave-one-out analysis indicated that the primary weight-gain effect was not driven by any single study. After sequential omission of each comparison, the pooled standardized effect remained positive and statistically significant, with estimates ranging from 0.68 to 1.02 and all corresponding *p*-values ≤ 0.006 (Supplementary Figure 1A).

For the primary MD analysis of weight-gain velocity, visual inspection of the funnel plot suggested asymmetry, and formal small-study-effect tests were statistically significant (Egger test beta = 10.83, SE = 2.775, *z* = 3.90, *P* < 0.001; Begg test *z* = 2.33, *P* = 0.020). In the exploratory trim-and-fill analysis, 2 studies were imputed on the left side of the funnel plot; the pooled estimate was attenuated after adjustment (observed MD 5.55 g/day, 95% CI 1.71–9.38; trim-and-fill adjusted MD 3.45, 95% CI −1.67 to 8.56) (Supplementary Figures 1B,C). These findings suggest possible small-study effects and potential overestimation of the primary effect size. Because the adjusted confidence interval included the null, the magnitude and robustness of the weight-gain effect remain uncertain. The trim-and-fill result was interpreted as a sensitivity analysis rather than as a definitive correction for publication bias.

## Discussion

This systematic review and meta-analysis synthesized randomized evidence on parent-administered massage-based interventions for preterm and low-birth-weight infants. Overall, massage-based interventions were associated with greater weight-gain velocity, higher odds of breastfeeding or human-milk feeding at discharge, shorter time to full oral feeding, and shorter hospital length of stay. Findings for parent-related psychological outcomes were generally favorable but varied across constructs, instruments, and assessment time points. In contrast, effects on length growth velocity, head-circumference growth, and motor development were not statistically significant. These findings suggest that parent-administered massage-based interventions may mainly influence short-term feeding and hospitalization outcomes, whereas evidence for structural growth and longer-term neurodevelopment remains less certain.

The most reliable findings were observed for selected feeding outcomes and hospital length of stay, for which the certainty of evidence was moderate. Although the pooled analysis suggested greater weight-gain velocity, this finding was supported by low-certainty evidence and should be considered less definitive. Parent-related psychological findings were also of low certainty because of substantial clinical and measurement heterogeneity. Evidence for length and head-circumference growth was of very low certainty, and motor-development findings were inconclusive.

The observed association with greater weight-gain velocity may be clinically relevant because early postnatal growth reflects nutritional adequacy, physiological stability, and discharge readiness (Ehrenkranz et al., 2006). This finding is consistent with previous research on massage-based interventions in preterm infants and with mechanistic studies suggesting that tactile or kinesthetic stimulation may increase vagal activity, improve gastric motility, and support nutrient utilization (Diego et al., 2007; Field et al., 1986; Field, 2010). However, the magnitude of the weight-gain effect should be interpreted with considerable caution. Between-study heterogeneity was very high (I^2^ = 97.87%), and both Egger’s and Begg’s tests indicated possible small-study effects. Moreover, the trim-and-fill analysis attenuated the pooled estimate from 5.55 g/day to 3.45 g/day, and the adjusted confidence interval included the null. These findings suggest that the observed weight-gain benefit may have been overestimated. Differences in gestational age, baseline maturity, intervention timing and duration, outcome-assessment interval, use of oil or emollient, clinical setting, and accompanying intervention components may also have contributed to the variability (Fenton et al., 2017; Getaneh et al., 2024; Mollà-Casanova et al., 2023; Niemi, 2017; Zhang et al., 2023). Therefore, the current evidence does not establish a precise or robust magnitude of benefit for weight gain, and confirmation in larger, standardized trials is required.

Gestational age may modify the response to massage-based interventions. Exploratory meta-regression suggested a larger standardized weight-gain effect in studies with higher mean gestational age. However, because this analysis was based on aggregate study-level data, it cannot establish that more mature individual infants derive greater benefit. A clinically preferable comparison of infants born <32 versus ≥32 weeks could not be performed reliably because several trials enrolled infants spanning both gestational-age categories and did not report outcomes separately by these strata.

Statistical significance should not be interpreted as equivalent to clinical importance. The included trials did not prespecify common minimal clinically important differences for weight-gain velocity, time to full oral feeding, or hospital length of stay. Clinical interpretation should therefore consider the absolute magnitude of the effect, confidence intervals, heterogeneity, certainty of evidence, and relevance to neonatal care. The reductions in time to full oral feeding and hospital length of stay may be clinically meaningful because these outcomes directly influence discharge readiness and were supported by moderate-certainty evidence. In contrast, the clinical importance of the weight-gain estimate remains uncertain because of very high heterogeneity, possible small-study effects, and attenuation after trim-and-fill adjustment.

The absence of statistically significant effects on length and head-circumference growth may be explained by several factors. Weight gain is a more responsive short-term marker than skeletal or cranial growth, which may require longer follow-up to show measurable differences. In addition, length and head-circumference measurements in preterm infants are vulnerable to measurement variability, which may reduce precision and statistical power (Fenton et al., 2017; Niemi, 2017). Therefore, the findings should not be interpreted as evidence that massage has no effect on broader growth trajectories, but rather that current evidence is insufficient to establish consistent effects on skeletal or cranial growth.

Because the included trials span several decades, we also explored whether the accumulated evidence changed over calendar time. In a cumulative meta-analysis ordered by publication year, early estimates were imprecise, whereas the cumulative evidence became more consistently positive as later studies were added, ultimately yielding the overall pooled benefit in weight-gain velocity. However, the pattern was not monotonic, and this analysis should not be interpreted as demonstrating that the effect of massage increased over time. More importantly, publication year cannot be considered a proxy for Family Integrated Care (FICare). Formal FICare implementation has varied across countries and institutions, and the included trials did not consistently report whether their NICUs operated within a formal FICare model. Changes over calendar time may also reflect concurrent evolution in neonatal care, skin-to-skin practices, parental participation, feeding support, study populations, and intervention protocols. Accordingly, the temporal analysis describes the evolution of the accumulated evidence but cannot determine whether FICare modified the effect of massage-based interventions.

Feeding and hospital outcomes were among the most clinically meaningful findings. Massage-based interventions were associated with faster transition to full oral feeding, higher odds of breastfeeding or human-milk feeding at discharge, and shorter hospital stay. These outcomes are closely linked in neonatal practice: improved behavioral organization, autonomic regulation, gastrointestinal function, and feeding readiness may accelerate oral feeding milestones and contribute to earlier discharge (Jadcherla et al., 2010; Sandnes et al., 2024; Seiiedi-Biarag and Mirghafourvand, 2020; World Health Organization, 2019; Zhang et al., 2023). However, the components included in these interventions may operate through different mechanisms. Oral stimulation may directly support sucking coordination and oral-motor readiness, whereas massage may primarily influence autonomic regulation, gastric motility, behavioral organization, and parent–infant interaction. Because several protocols combined massage with oral stimulation, kinesthetic stimulation, visual interaction, kangaroo care, or structured parent training, the feeding and hospitalization findings cannot be attributed solely to massage. They are more appropriately interpreted as effects of parent-delivered developmental-care packages that included massage as one component. It should be noted that, the pooled feeding-at-discharge outcome should also be interpreted cautiously because the contributing trials used somewhat different definitions, including direct breastfeeding and human-milk feeding, and did not consistently distinguish exclusive from non-exclusive feeding. The pooled estimate therefore reflects the feeding definitions used by the individual trials.

Intervention heterogeneity is a central limitation of this review. Some trials evaluated massage-focused protocols, whereas others assessed multimodal interventions combining massage with oral, tactile, kinesthetic, visual, or developmental-care components. These components differ in their intended targets and mechanisms and may contribute differently to feeding, growth, behavioral, and parental outcomes. Consequently, the pooled estimates represent the average effects of a heterogeneous group of parent-administered interventions that included massage and should not be interpreted as estimates of the independent effect of massage alone.

Skin-to-skin care and parent-administered massage share several features, including parental proximity, physical contact, and active parent involvement, and both may contribute to physiological and behavioral regulation. An additional consideration is whether the observed effects are attributable specifically to massage or partly to increased parental presence and interaction. Parent-administered massage necessarily involves parental proximity, physical contact, focused attention, and an active caregiving role, each of which may independently influence infant behavioral regulation and parent-related outcomes. In many included trials, these components were not experimentally separated, and therefore the pooled estimates cannot isolate the effect of massage from the broader effects of increased parental involvement. Some studies provide partial separation of these effects. For example, in the study by Mendes and Procianoy (2008) and its follow-up by Procianoy et al. (2010), both intervention and control groups received kangaroo care, while only the intervention group received additional maternal tactile–kinesthetic stimulation; these trials therefore estimate the incremental effect of tactile–kinesthetic stimulation beyond skin-to-skin contact. Nevertheless, across the evidence base as a whole, the independent contributions of massage and parental presence remain uncertain (Mendes and Procianoy, 2008; Procianoy et al., 2010).

Evidence for motor development was limited and statistically non-significant. The pooled estimate favored massage, but confidence intervals crossed the null. This may reflect the small number of trials, differing developmental instruments, short or variable follow-up, and heterogeneity in intervention content. Several biologically plausible mechanisms may contribute to the observed short-term effects of massage-based interventions. In preterm infants, tactile or kinesthetic stimulation has been associated with increased vagal activity and gastric motility, which may facilitate digestion, nutrient utilization, and weight gain. Tactile input may also influence behavioral organization, arousal, and stress regulation, although evidence for consistent changes in physiological stress markers remains limited. Experimental animal studies provide additional evidence for experience-dependent neuroplasticity. In infant rats with early cortical injury, Kolb and Gibb found that repeated tactile stimulation reduced subsequent behavioral impairment and produced region-specific changes in dendritic length and spine density (Kolb and Gibb, 2010). In normally developing rats, Richards et al. (2012) similarly reported that early-life tactile stimulation altered later behavior and increased dendritic branching, dendritic length, and spine density in examined prefrontal and amygdalar regions. These experimental findings support the biological plausibility that repeated tactile experience can influence the developing nervous system; however, they represent indirect mechanistic evidence and should not be extrapolated to suggest that massage produces equivalent neurodevelopmental effects in human preterm infants. Consistent with this distinction, the present meta-analysis did not demonstrate a statistically significant improvement in motor development (Orton et al., 2024). Future trials should include standardized developmental assessments and follow-up beyond discharge and infancy.

Parent-related psychological outcomes were not combined into a single pooled effect because the included studies evaluated different constructs, including parental stress, anxiety, mood disturbance, attachment, perceived competence, and caregiving self-efficacy. The studies also used different instruments and assessment time points, including assessments during hospitalization, at discharge, and after discharge. Findings generally favored the intervention in some domains, and parent-administered massage may provide a structured caregiving role that supports parental involvement and perceived competence. However, the magnitude and consistency of these effects remain uncertain. Future trials should define the intended psychological construct *a priori*, use validated domain-specific instruments, and standardize assessment timing. Stress, anxiety, mood symptoms, attachment, and self-efficacy should be analyzed as separate outcome domains rather than combined into a single summary estimate (Sandnes et al., 2024; Shoghi et al., 2018; Treyvaud, 2014; World Health Organization, 2018).

The subgroup and meta-regression analyses should be considered exploratory and hypothesis-generating. Although study-level characteristics including oil use, intervention provider, gestational age, timing of initiation, and session duration showed potential associations with the weight-gain effect, several analyses were based on very few studies, and all were based on aggregate study-level rather than individual-participant data. These findings are therefore vulnerable to ecological bias, confounding, and model instability and should not be used to guide current clinical practice or define an optimal intervention protocol. They may instead help identify variables for prospective evaluation in adequately powered comparative trials or individual-participant-data analyses.

Notably, the provider analysis suggested positive weight-gain effects in both mother-only interventions and interventions that could be delivered by either or both parents, with no statistically significant difference between these provider categories. However, the limited number of studies and sparse representation of fathers preclude conclusions regarding whether intervention effectiveness differs by specific parental provider.

This review has several strengths. It focused on parent-administered massage-based interventions, incorporated both infant- and parent-centered outcomes, and used random-effects meta-analysis, sensitivity analysis, subgroup analysis, meta-regression, risk-of-bias assessment, and GRADE evaluation. It also integrated recent randomized trials and evaluated sources of clinical heterogeneity.

Several limitations should be acknowledged. First, many interventions were multimodal, limiting attribution of effects to massage alone. Second, intervention protocols differed substantially in timing, dose, duration, provider training, oil use, and setting. Third, blinding of participants and caregivers was generally not feasible, creating some risk-of-bias concerns. Fourth, several subgroup and meta-regression analyses included few studies and relied on aggregate study-level characteristics, limiting statistical stability and preventing inference regarding treatment-effect modification at the individual-patient level. Fifth, the primary weight-gain outcome showed very high heterogeneity and evidence of possible small-study effects, and the trim-and-fill adjusted estimate was attenuated and no longer statistically significant. Sixth, parent-related psychological outcomes differed substantially in the constructs, instruments, scoring directions, and assessment time points used, precluding a clinically meaningful common pooled estimate. Seventh, assessment intervals for growth outcomes varied across studies. Although changes were normalized to daily growth velocities using the corresponding study-specific intervals, differences in the duration and timing of assessment may still have contributed to between-study heterogeneity.

In summary, the evidence was strongest for selected feeding outcomes and hospital length of stay, for which the certainty of evidence was moderate. Evidence for greater weight-gain velocity was less certain because of very high heterogeneity, low certainty, possible small-study effects, and attenuation after trim-and-fill adjustment. Parent-related psychological findings were also uncertain because of substantial variation in the constructs, instruments, and assessment time points used. Current evidence does not establish consistent benefits for length growth, head-circumference growth, or motor development. Because many interventions combined massage with other developmental-care components, the observed effects should be interpreted as those of parent-delivered developmental-care packages that include massage rather than massage alone. Large, adequately powered trials using standardized protocols, clearly defined comparators, consistent outcome measures, transparent adherence reporting, and longer follow-up are required.

## Conclusion

Moderate-certainty evidence suggests that parent-administered massage-based interventions may improve breastfeeding or human-milk feeding at discharge, shorten time to full oral feeding, and reduce hospital length of stay in preterm or low-birth-weight infants. Evidence for greater weight-gain velocity remains uncertain because of very high heterogeneity, low certainty, possible small-study effects, and attenuation of the estimate after trim-and-fill adjustment. Parent-related psychological findings are also supported by low-certainty evidence because of substantial clinical and measurement heterogeneity. Evidence remains insufficient to establish consistent benefits for length growth, head-circumference growth, or motor development. Because many included interventions combined massage with oral, kinesthetic, tactile, skin-to-skin, or other developmental-care components, the observed findings support parent-delivered developmental-care packages that include massage rather than the independent effectiveness of massage alone. Standardized, adequately powered trials with longer follow-up are needed to clarify the independent and sustained effects of massage and to define optimal implementation within family-centered neonatal care.

## Data Availability

The original contributions presented in this study are included in this article/Supplementary material, further inquiries can be directed to the corresponding author.
