# Supplementary material for: Effects of parent-administered massage-based multimodal interventions on health outcomes in preterm infants: a systematic review and meta-analysis
Source: Front Psychol. 2026 Oct 2;17:1922153. doi: 10.3389/fpsyg.2026.1922153 (PMC13672638; doi:10.3389/fpsyg.2026.1922153)
Supplement: Supplementary file 1 [file Supplementary_file_1.docx]

**Supplementary table S1. Keywords used for search in different databases**

| **Keywords for Premature / Low Birth Weight Infants** | **Keywords for Massage / Touch Interventions** | **Keywords for Growth, Development, and Maternal Outcomes** |
| --- | --- | --- |
| Infant, Premature, Infant, Extremely Premature, Infant, Low Birth Weight, Infant, Very Low Birth Weight, preterm infant*, premature infant*, preterm neonate*, premature neonate*, preterm newborn*, premature newborn*, neonatal prematurity, prematurity, low birth weight, very low birth weight, extremely low birth weight, LBW, VLBW, ELBW | Massage, Massage Therapy, Touch, Tactile Perception, infant massage, massage therapy, massage therapies, tactile kinesthetic stimulation, kinesthetic stimulation, tactile stimulation, sensory stimulation, parent-administered massage, parent delivered massage, parent-mediated massage, maternal massage, mother-administered massage, mother delivered massage, touch therapy, therapeutic touch, gentle touch, affective touch, skin-to-skin, skin-to-skin contact, kangaroo care, kangaroo mother care | Growth and Development, Body Weight, Body Height, Birth Weight, Head Circumference, Anthropometry, Body Composition, growth, physical growth, somatic growth, growth velocity, growth rate, catch-up growth, developmental growth, body weight, weight gain, daily weight gain, weight increment, weight change*, body length, infant length, crown-heel length, linear growth, height gain, head circumference, occipitofrontal circumference, chest circumference, thoracic circumference, abdominal circumference, abdomen circumference, arm circumference, biceps circumference, thigh circumference, anthropometr*, body composition, child development, infant development, neonatal development, neurodevelopment*, neurodevelopmental outcome*, developmental outcome*, cognitive development, motor development, psychomotor development, neuromotor development, psychomotor performance, reflex*, reflex status, neonatal reflex*, primitive reflex*, Bayley, Bayley Scales, Bayley Scales of Infant Development, Bayley-III, BSID, bonding, attachment, attachment behavior, maternal attachment, mother-infant attachment, parent-infant attachment, mother-infant bonding, parent-infant bonding, maternal-infant bonding, mother-infant interaction, parent-infant interaction, maternal stress, parental stress, psychological stress, stress response, anxiety, maternal anxiety, postpartum anxiety, depression, depressive symptoms, depressive symptom, emotional depression, maternal depression, postpartum depression, postnatal depression, mental health, maternal mental health, cortisol, salivary cortisol, serum cortisol, stress hormone* |

Search terms were combined within each concept using OR and across concepts using AND. The asterisk (*) denotes truncation or wildcard searching where supported by the database; **Abbreviations:** BSID, Bayley Scales of Infant Development; ELBW, extremely low birth weight; LBW, low birth weight; MeSH, Medical Subject Headings; VLBW, very low birth weight.

**Supplementary table S2. Search strategy and the associated results in different databases**

| **Database** | **Search Strategy** | **Results** |
| --- | --- | --- |
| PubMed | ("Infant, Premature"[Mesh] OR "Infant, Extremely Premature"[Mesh] OR "Infant, Low Birth Weight"[Mesh] OR "Infant, Very Low Birth Weight"[Mesh] OR preterm infant* OR premature infant* OR preterm neonate* OR premature neonate* OR preterm newborn* OR premature newborn* OR neonatal prematurity OR prematurity OR low birth weight OR very low birth weight OR extremely low birth weight OR LBW OR VLBW OR ELBW) AND ("Massage"[Mesh] OR "Massage Therapy"[Mesh] OR "Touch"[Mesh] OR "Tactile Perception"[Mesh] OR infant massage OR massage therapy OR tactile stimulation OR sensory stimulation OR therapeutic touch OR skin-to-skin OR kangaroo care OR kangaroo mother care) AND ("Growth and Development"[Mesh] OR "Body Weight"[Mesh] OR growth OR weight gain OR anthropometry OR neurodevelopment* OR child development OR bonding OR attachment OR maternal stress OR anxiety OR depression OR cortisol) NOT (animals[mh] NOT humans[mh]) | 1,383 |
| Scopus | TITLE-ABS-KEY ( ( "Infant, Premature" OR "Infant, Extremely Premature" OR "Infant, Low Birth Weight" OR "Infant, Very Low Birth Weight" OR preterm infant* OR premature infant* OR preterm neonate* OR premature neonate* OR preterm newborn* OR premature newborn* OR neonatal prematurity OR prematurity OR "low birth weight" OR "very low birth weight" OR "extremely low birth weight" OR LBW OR VLBW OR ELBW ) AND ( Massage OR "Massage Therapy" OR Touch OR "Tactile Perception" OR "infant massage" OR "massage therapy" OR "massage therapies" OR "tactile kinesthetic stimulation" OR "kinesthetic stimulation" OR "tactile stimulation" OR "sensory stimulation" OR "parent-administered massage" OR "parent delivered massage" OR "parent-mediated massage" OR "maternal massage" OR "mother-administered massage" OR "mother delivered massage" OR "touch therapy" OR "therapeutic touch" OR "gentle touch" OR "affective touch" OR skin-to-skin OR "skin-to-skin contact" OR "kangaroo care" OR "kangaroo mother care" ) AND ( "Growth and Development" OR "Body Weight" OR "Body Height" OR "Birth Weight" OR "Head Circumference" OR Anthropometry OR "Body Composition" OR growth OR "physical growth" OR "somatic growth" OR "growth velocity" OR "growth rate" OR "catch-up growth" OR "developmental growth" OR "body weight" OR "weight gain" OR "daily weight gain" OR "weight increment" OR "weight change*" OR "body length" OR "infant length" OR "crown-heel length" OR "linear growth" OR "height gain" OR "birth weight" OR "head circumference" OR "occipitofrontal circumference" OR "chest circumference" OR "thoracic circumference" OR "abdominal circumference" OR "abdomen circumference" OR "arm circumference" OR "biceps circumference" OR "thigh circumference" OR anthropometr* OR "body composition" OR "Child Development" OR "Psychomotor Performance" OR "child development" OR "infant development" OR "neonatal development" OR neurodevelopment* OR "neurodevelopmental outcome*" OR "developmental outcome*" OR "cognitive development" OR "motor development" OR "psychomotor development" OR "neuromotor development" OR "psychomotor performance" OR reflex* OR "reflex status" OR "neonatal reflex*" OR "primitive reflex*" OR Bayley OR "Bayley Scales" OR "Bayley Scales of Infant Development" OR Bayley-III OR BSID OR "Mother-Child Relations" OR "Parent-Child Relations" OR "Attachment Behavior" OR bonding OR attachment OR "attachment behavior" OR "maternal attachment" OR "mother-infant attachment" OR "parent-infant attachment" OR "mother-infant bonding" OR "parent-infant bonding" OR "maternal-infant bonding" OR "mother-infant interaction" OR "parent-infant interaction" OR "Stress, Psychological" OR Anxiety OR Depression OR "Mental Health" OR "maternal stress" OR "parental stress" OR "psychological stress" OR "stress response" OR anxiety OR "maternal anxiety" OR "postpartum anxiety" OR depression OR "depressive symptoms" OR "depressive symptom" OR "emotional depression" OR "maternal depression" OR "postpartum depression" OR "postnatal depression" OR "mental health" OR "maternal mental health" OR Hydrocortisone OR cortisol OR "salivary cortisol" OR "serum cortisol" OR "stress hormone*" ) ) AND NOT TITLE-ABS-KEY ( animal* AND NOT human* ) | 1,101 |
| Web of Science | TS=(("Infant, Premature" OR "Infant, Extremely Premature" OR "Infant, Low Birth Weight" OR "Infant, Very Low Birth Weight" OR preterm infant* OR premature infant* OR preterm neonate* OR premature neonate* OR preterm newborn* OR premature newborn* OR neonatal prematurity OR prematurity OR low birth weight OR very low birth weight OR extremely low birth weight OR LBW OR VLBW OR ELBW) AND (Massage OR "Massage Therapy" OR Touch OR "Tactile Perception" OR infant massage OR massage therapy OR massage therapies OR tactile kinesthetic stimulation OR kinesthetic stimulation OR tactile stimulation OR sensory stimulation OR parent-administered massage OR parent delivered massage OR parent-mediated massage OR maternal massage OR mother-administered massage OR mother delivered massage OR touch therapy OR therapeutic touch OR gentle touch OR affective touch OR skin-to-skin OR skin-to-skin contact OR kangaroo care OR kangaroo mother care) AND ("Growth and Development" OR "Body Weight" OR "Body Height" OR "Birth Weight" OR "Head Circumference" OR Anthropometry OR "Body Composition" OR growth OR physical growth OR somatic growth OR growth velocity OR growth rate OR catch-up growth OR developmental growth OR body weight OR weight gain OR daily weight gain OR weight increment OR weight change* OR body length OR infant length OR crown-heel length OR linear growth OR height gain OR birth weight OR head circumference OR occipitofrontal circumference OR chest circumference OR thoracic circumference OR abdominal circumference OR abdomen circumference OR arm circumference OR biceps circumference OR thigh circumference OR anthropometr* OR body composition OR "Child Development" OR "Psychomotor Performance" OR child development OR infant development OR neonatal development OR neurodevelopment* OR neurodevelopmental outcome* OR developmental outcome* OR cognitive development OR motor development OR psychomotor development OR neuromotor development OR psychomotor performance OR reflex* OR reflex status OR neonatal reflex* OR primitive reflex* OR Bayley OR "Bayley Scales" OR "Bayley Scales of Infant Development" OR Bayley-III OR BSID OR "Mother-Child Relations" OR "Parent-Child Relations" OR "Attachment Behavior" OR bonding OR attachment OR attachment behavior OR maternal attachment OR mother-infant attachment OR parent-infant attachment OR mother-infant bonding OR parent-infant bonding OR maternal-infant bonding OR mother-infant interaction OR parent-infant interaction OR "Stress, Psychological" OR Anxiety OR Depression OR "Mental Health" OR maternal stress OR parental stress OR psychological stress OR stress response OR anxiety OR maternal anxiety OR postpartum anxiety OR depression OR depressive symptoms OR depressive symptom OR emotional depression OR maternal depression OR postpartum depression OR postnatal depression OR mental health OR maternal mental health OR Hydrocortisone OR cortisol OR salivary cortisol OR serum cortisol OR stress hormone*)) NOT TS=(animal* NOT human*) | 2,240 |

**Supplementary Table S3. Risk-of-bias assessment of included randomized controlled trials using the Cochrane RoB 2 tool.**

| **Study (Author, Year)** | **Domain 1: Bias arising from the randomization process** | **Domain 2: Bias due to deviations from intended interventions** | **Domain 3: Bias due to missing outcome data** | **Domain 4: Bias in measurement of the outcome** | **Domain 5: Bias in selection of the reported result** | **Overall risk of bias** |
| --- | --- | --- | --- | --- | --- | --- |
| Fan et al. (2021) | Low risk of bias | Low risk of bias | Low risk of bias | Low risk of bias | Low risk of bias | Low risk of bias |
| Fucile et al. (2025) | Low risk of bias | Some concerns | Low risk of bias | Some concerns | Low risk of bias | Some concerns |
| Kumar et al. (2013) | Low risk of bias | Some concerns | Low risk of bias | Some concerns | Low risk of bias | Some concerns |
| Lotfalipour et al. (2019) | Some concerns | Some concerns | Low risk of bias | Some concerns | Low risk of bias | Some concerns |
| Keshavarz et al. (2012) | Low risk of bias | Some concerns | Low risk of bias | Low risk of bias | Low risk of bias | Low risk of bias |
| Fontana et al. (2018) | Low risk of bias | Low risk of bias | Low risk of bias | Low risk of bias | Low risk of bias | Low risk of bias |
| Fontana et al. (2020) | Low risk of bias | Some concerns | Low risk of bias | Low risk of bias | Low risk of bias | Low risk of bias |
| Hwu et al. (2023) | Low risk of bias | Some concerns | Low risk of bias | Some concerns | Low risk of bias | Some concerns |
| Jamshaid et al. (2021) | Low risk of bias | Some concerns | Low risk of bias | Some concerns | Low risk of bias | Some concerns |
| Pramitha et al. (2026) | Low risk of bias | Some concerns | Low risk of bias | Some concerns | Low risk of bias | Some concerns |
| Alijanzadeh Zaferani et al. (2021) | Low risk of bias | Some concerns | Low risk of bias | Some concerns | Low risk of bias | Some concerns |
| Lai et al. (2022) | Low risk of bias | Some concerns | Low risk of bias | Low risk of bias | Low risk of bias | Low risk of bias |
| Procianoy et al. (2010) | Low risk of bias | Some concerns | Low risk of bias | Low risk of bias | Low risk of bias | Low risk of bias |
| Zhang et al. (2019) | Low risk of bias | Some concerns | Low risk of bias | Low risk of bias | Low risk of bias | Low risk of bias |
| Holditch-Davis et al. (2014) | Low risk of bias | Some concerns | Low risk of bias | Some concerns | Low risk of bias | Some concerns |
| Mendes & Procianoy (2008) | Low risk of bias | Some concerns | Low risk of bias | Low risk of bias | Low risk of bias | Low risk of bias |
| White-Traut & Nelson (1988) | Low risk of bias | Some concerns | Low risk of bias | Some concerns | Low risk of bias | Some concerns |
| Matricardi et al. (2013) | Low risk of bias | Some concerns | Low risk of bias | Some concerns | Low risk of bias | Some concerns |
| Feijó et al. (2006) | Low risk of bias | Some concerns | Low risk of bias | Some concerns | Low risk of bias | Some concerns |
| González et al. (2023) | Low risk of bias | Some concerns | Low risk of bias | Low risk of bias | Low risk of bias | Low risk of bias |
| Fallah et al. (2013) | Low risk of bias | Some concerns | Low risk of bias | Low risk of bias | Low risk of bias | Low risk of bias |
| Whipple (2000) | Low risk of bias | Some concerns | Low risk of bias | Some concerns | Low risk of bias | Some concerns |
| Shoghi et al. (2018) | Low risk of bias | Some concerns | Low risk of bias | Some concerns | Low risk of bias | Some concerns |
| Gonzalez et al. (2009) | Low risk of bias | Some concerns | Low risk of bias | Low risk of bias | Low risk of bias | Low risk of bias |

Domain-level and overall judgments were made according to the revised Cochrane Risk of Bias 2 tool. Green cells indicate low risk of bias; yellow cells indicate some concerns; Abbreviations: RoB 2, revised Cochrane Risk of Bias 2 tool; RCT, randomized controlled trial.

**Supplementary Table** **S4.** **GRADE certainty of evidence for parent-administered massage-based interventions compared with standard care or non-massage controls.**

| **Outcome** | **Evidence base** | **Risk of bias** | **Inconsistency** | **Indirectness** | **Imprecision** | **Publication bias** | **Certainty** |
| --- | --- | --- | --- | --- | --- | --- | --- |
| Weight-gain velocity (primary) | 10 RCTs; pooled as MD in g/day; additional subgroup, cumulative, sensitivity, and meta-regression analyses were performed for this primary outcome. | Some concerns: several trials had unavoidable lack of participant/caregiver blinding and concerns about outcome measurement or deviations from intended interventions. | Serious: very high heterogeneity (I2=97.9%) and marked variation in protocols, oil use, setting, duration, and outcome timing. | Not serious to some concerns: population and outcome were directly relevant, but several interventions were multimodal rather than massage alone. | Not serious for direction of effect, although the width of the interval and heterogeneity reduce confidence in magnitude. | Suspected: funnel-plot asymmetry; Egger p<0.001; trim-and-fill attenuated the pooled effect. | Low |
| Length growth velocity | 5 RCTs; pooled as MD in cm/day. | Some concerns: blinding limitations and some outcome-measurement concerns. | Serious: substantial heterogeneity (I2=86.7%). | Not serious: outcome directly reflects somatic growth in preterm infants. | Serious: confidence interval crossed the null and was compatible with minimal or no effect. | Undetected/ difficult to assess because of few studies. | Very low |
| Head-circumference growth | 7 RCTs; pooled as MD in cm/day. | Some concerns: blinding and measurement issues in some studies. | Some concerns: moderate heterogeneity (I2=51.7%) with variable timing and protocols. | Not serious: outcome directly reflects cranial growth. | Serious: confidence interval crossed the null and the observed effect was negligible. | Undetected/ difficult to assess because of few studies. | Very low |
| Breastfeeding or human-milk feeding at discharge | 2 RCTs; dichotomous outcome reported as OR. | Some concerns: intervention delivery could not be blinded. | Not serious: heterogeneity was absent (I2=0%). | Some concerns: outcome combined direct breastfeeding and human-milk feeding definitions across studies. | Serious: only two studies, although the effect estimate was large and statistically significant. | Undetected/ difficult to assess because of very few studies. | Moderate |
| Time to full oral feeding | 3 RCTs; pooled as MD in days. | Some concerns: blinding limitations and variable co-interventions. | Some concerns: moderate heterogeneity (I2=46.8%). | Some concerns: some interventions included oral/sensorimotor components in addition to massage. | Some concerns: confidence interval was close to the null, but the point estimate was clinically favorable. | Undetected/ difficult to assess because of few studies. | Moderate |
| Hospital length of stay | 3 RCTs; pooled as MD in days. | Some concerns: unavoidable lack of blinding and some intervention-delivery concerns. | Not serious: heterogeneity was absent (I2=0%). | Some concerns: discharge can be influenced by local clinical practice and feeding policies. | Not serious: confidence interval excluded the null and the reduction was clinically meaningful. | Undetected/ difficult to assess because of few studies. | Moderate |
| Motor development | 3 RCTs; pooled as SMD because different developmental scales were used. | Some concerns: outcome assessment and intervention-delivery limitations in some studies. | Not serious: heterogeneity was absent (I2=0%). | Some concerns: developmental tools and follow-up intervals differed across studies. | Serious: confidence interval crossed the null and the number of studies was small. | Undetected/ difficult to assess because of few studies. | Low |
| Parental stress or mood disturbance | 4 RCTs; pooled as MD where comparable scoring direction could be harmonized. | Some concerns: participant blinding was impossible and self-reported outcomes are susceptible to expectation effects. | Very serious: extremely high heterogeneity (I2=99%) across scales, time points, and settings. | Some concerns: instruments assessed related but not identical psychological constructs. | Some concerns: statistically significant pooled effect, but magnitude is uncertain because of heterogeneity. | Undetected/ difficult to assess because of few studies. | Low |

Evidence from randomized trials was initially rated as high certainty and downgraded for risk of bias, inconsistency, indirectness, imprecision, or publication bias when applicable. Effect estimates favor massage-based interventions when positive for growth, breastfeeding/human-milk feeding, and motor development, and when negative for time to full oral feeding, hospital length of stay, and parental stress/mood disturbance. Abbreviations: CI, confidence interval; GRADE, Grading of Recommendations Assessment, Development and Evaluation; MD, mean difference; OR, odds ratio; RoB, risk of bias; SMD, standardized mean difference.

**Supplementary figures and captions**

Supplementary Figure S1. Sensitivity and small-study-effect diagnostics for the primary weight-gain outcome. (A) Leave-one-out sensitivity analysis using standardized weight-gain effects. (B) Funnel plot for the mean-difference analysis of weight-gain velocity. (C) Trim-and-fill funnel plot for the mean-difference analysis, showing observed and imputed studies.

Supplementary Figure S2. Cumulative meta-analysis of weight-gain velocity ordered by publication year. Studies were entered chronologically according to publication year, and the pooled random-effects estimate was recalculated after the addition of each study. Mean differences are expressed as g/day with 95% confidence intervals. This analysis illustrates how the cumulative evidence for weight-gain velocity evolved over time and should not be interpreted as a direct assessment of the effect of Family Integrated Care implementation.
